# Optimizing outcomes in total knee arthroplasty: the role of patellar resurfacing and tibial ınsert type

**DOI:** 10.1590/1806-9282.20250101

**Published:** 2025-10-17

**Authors:** Sönmez Sağlam, Zekeriya Okan Karaduman, Mehmet Arıcan, Mücahid Osman Yücel, Raşit Emin Dalaslan, Mehmet Akif Köse, Veysel Uludağ

**Affiliations:** 1 Duzce University, Faculty of Medicine, Department of Orthopaedics and Traumatology – Düzce, Türkiye.; 2 Şişli Hamidiye Etfal Training And Research Hospital – Istanbul, Türkiye.; 3 Düzce University, Faculty of Health Sciences, Department of Physiotherapy and Rehabilitation – Düzce, Türkiye.

**Keywords:** Total knee arthroplasty, Knee osteoarthritis, Prosthesis design, Patellofemoral joints

## Abstract

**OBJECTIVE:**

Total knee arthroplasty is a common procedure for advanced knee osteoarthritis, aiming to reduce pain and restore function. However, the impact of patellar resurfacing and tibial insert types (fixed vs. mobile) on clinical outcomes remains debated, with limited comparative studies.

**METHODS:**

This retrospective cohort study included total knee arthroplasty patients (2012–2018) with ≥5 years of follow-up, divided into four groups based on insert type and resurfacing status. Clinical outcomes were assessed using visual analog scale, Western Ontario and McMaster Universities Osteoarthritis Index, timed up and go, and range of motion. Shapiro-Wilk tests were used to assess normality, and group comparisons were conducted using non-parametric statistical methods.

**RESULTS:**

Non-resurfacing groups had significantly higher pain scores (p<0.001). Mobile inserts provided better flexion range of motion and Western Ontario and McMaster Universities Osteoarthritis Index functional scores than fixed inserts (p<0.001). The best functional outcomes were observed in the mobile insert with the resurfacing group. A significant correlation was found between timed up and go and Western Ontario and McMaster Universities Osteoarthritis Index total scores in the fixed insert without resurfacing group (r=0.424, p=0.008), while no such correlation was observed in other groups.

**CONCLUSION:**

Patellar resurfacing and mobile tibial inserts enhance pain relief, mobility, and function in total knee arthroplasty patients. However, due to the retrospective nature of the study and group heterogeneity, prospective multicenter trials are warranted to validate these findings. These findings emphasize the importance of individualized implant selection, warranting further prospective, multicenter studies.

## INTRODUCTION

Total knee arthroplasty (TKA) is a well-established procedure to relieve pain, improve joint mobility, and enhance quality of life in advanced knee osteoarthritis^1,2^. With the aging population, its prevalence has increased, but long-term success depends on factors like implant selection, surgical technique, and patellar resurfacing decisions^3^.

Patellar resurfacing replaces the patellar surface during TKA to reduce anterior knee pain, which is common in non-resurfaced cases. While early prostheses did not routinely include resurfacing, their potential benefits have gained interest. However, it remains controversial due to complications such as malalignment, loosening, and fracture^4,5^. Some studies report improved pain and function, especially for stair-related activities, while others question its long-term benefits^6,7^. More recent meta-analyses, however, support selective resurfacing in patients with severe patellofemoral osteoarthritis (PF OA)^8^.

Tibial insert type is another key factor in TKA outcomes. Fixed inserts provide stability, whereas mobile inserts allow greater movement and better load distribution. Some studies suggest mobile inserts improve range of motion and function, while others find no significant advantage^9,10^. Biomechanical studies have proposed that mobile inserts may better accommodate rotational forces and reduce peak stresses at the bone-implant interface^11,12^.

Although both factors have been studied independently, few studies have evaluated their combined effects on clinical outcomes. Previous research has often been limited by short follow-ups, small sample sizes, or a lack of focus on specific patient populations. Moreover, methodological heterogeneity, such as inconsistent criteria for resurfacing and lack of adjustment for patient-related confounders, has made comparisons difficult.

This study aims to address these gaps by analyzing the effects of patellar resurfacing and tibial insert type on pain (visual analog scale [VAS]), function (WOMAC, timed up and go [TUG]), and range of motion over a minimum 5-year follow-up. In addition, this study incorporates correlation and subgroup analyses to better understand the biomechanical relationships between implant characteristics and clinical performance.

We hypothesize that combining patellar resurfacing with mobile tibial inserts yields superior clinical and functional outcomes. This study seeks to provide evidence-based insights to guide clinical decision-making in TKA.

## METHODS

This retrospective cohort study included patients who underwent TKA between 2012 and 2018 at Duzce University Faculty of Medicine, Orthopedics and Traumatology Department, with a minimum 5-year follow-up. A total of 147 patients were categorized into four groups based on tibial insert type (fixed or mobile) and patellar resurfacing status (resurfaced or non-resurfaced). All surgeries were performed by high-volume orthopedic surgeons using standardized protocols to minimize surgeon-dependent variability.

**Figure 1:**
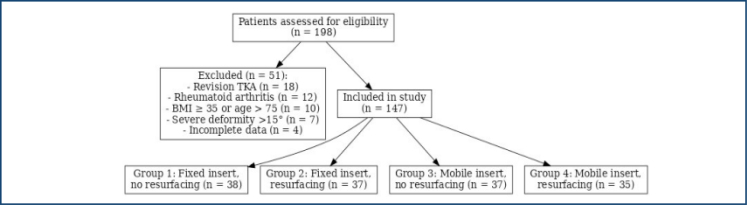
Flow diagram of patient selection and group allocation.

The study design, including patient inclusion, exclusion criteria, and group allocation, is summarized in Figure 1.

### Surgical technique

All patients underwent standardized TKA via a midline incision and medial parapatellar approach. Both cruciate ligaments were resected, with distal femoral and proximal tibial cuts at 5°–7° and 4°–7° angles, respectively. All received PNG (Pasifik, Ankara, Türkiye) implants with cruciate-sacrificing prostheses.

The groups were defined as follows: The first group consisted of patients who received fixed tibial inserts without patellar resurfacing. The second group included patients who received fixed tibial inserts with patellar resurfacing. The third group comprised patients who received mobile tibial inserts without patellar resurfacing. The fourth group included patients who received mobile tibial inserts with patellar resurfacing. Patellar resurfacing was performed in cases with severe PF OA (grade 4), while in grades 1–3, the decision was left to the surgeon's discretion based on intraoperative findings.

### Inclusion and exclusion criteria

Inclusion criteria: Age ≤75 years, body mass index (BMI) <35 kg/m^2^, primary TKA with documented tibial insert and patellar resurfacing status, and ≥5-year follow-up. Preoperative assessments included PF OA grading and lower limb alignment.

Exclusion criteria: Secondary indications (e.g., rheumatoid/post-traumatic arthritis), revision surgery, cognitive impairment, insufficient follow-up data, or preoperative valgus/varus deformities >15°.

### Preoperative and patient characteristics

Radiographic assessments included Kellgren-Lawrence grading for PF OA and hip-knee-ankle angle for limb alignment. Severe PF OA (grade 4) indicated patellar resurfacing, while grades 1–3 were evaluated at the surgeon's discretion. Baseline WOMAC and VAS scores ensured group comparability. Patient data were cross-checked for completeness and consistency using electronic medical records.

### Ethical approval

Ethical approval was obtained from the Duzce University Ethics Committee (Decision No.: 2024/224, Date: 04/11/2024). Written informed consent was obtained, and the study followed the Declaration of Helsinki and good clinical practice guidelines. Data were anonymized before analysis.

### Evaluation parameters

Evaluation parameters included pain assessment using the VAS (0–10), functionality measured by the WOMAC Index (pain, stiffness, and function), and the TUG test. Range of motion (ROM) was evaluated through knee flexion and extension measurements using a goniometer. Measurements were performed by experienced physiotherapists blinded to the implant type to reduce observer bias. Additionally, demographic data such as age, gender, BMI, surgical side, and follow-up duration were recorded.

## RESULTS

A total of 147 patients were included in the study, categorized into four groups: 38 patients with a fixed tibial insert without patellar resurfacing, 37 with a fixed insert and patellar resurfacing, 37 with a mobile insert without patellar resurfacing, and 35 with a mobile insert and patellar resurfacing.

The demographic and clinical characteristics of these groups, including age, bBMI, follow-up duration, gender distribution, and side of the operated knee, were statistically analyzed. There were no significant differences among the groups in terms of these parameters (p>0.05).

The mean age of patients ranged from approximately 67.7 to 69.9 years, with BMI values between 27.6 and 28.4 kg/m^2^. The average follow-up period varied from 70.5 to 73.2 months. Gender distribution was similar across the groups, with women comprising approximately 73 to 78% of each group. Likewise, the right and left knee distributions were comparable, with no statistically significant differences among the groups.

According to Table 1, significant differences were observed in VAS and WOMAC scores among the groups (p<0.001), as identified by the Kruskal-Wallis Multiple Comparison test.

**Table 1:** Comparison of visual analog scale and Western Ontario and McMaster Universities Osteoarthritis Index scores of patients with fixed insert and no patella resurfacing, fixed insert and patella resurfacing, mobile insert and no patella resurfacing, mobile insert and patella resurfacing^a^,^b^,^c^,^d^.

	Mean±SD; median (Min–Max)				p*
	Fixed insert^a^ and no patella resurfacing (n=38)	Fixed insert^b^ and patella resurfacing (n=37)	Mobile insert^c^ and no patella resurfacing (n=37)	Mobile insert^d^ and patella resurfacing (n=35)	
VAS score	2.53±0.95 3 (0–5)	1.30±0.87 1 (0–4)	2.57±1.11 3 (0–5)	1.60±1.14 2 (0–5)	<0.001^b^
p^bb^	**a–b p<0.001**, a–c p=1.000, **a–d p<0.001**, **b–c p<0.001**, b–d p=1.000, **c–d p=0.01**				
WOMAC pain score	6.29±1.08 6 (4–9)	2.73±1.01 3 (1–5)	5.78±1.22 6 (3–9)	2.20±0.96 2 (0–5)	<0.001^b^
p^bb^	**a–b p<0.001**, a–c p=1.000, **a–d p<0.001**, **b–c p<0.001**, b–d p=1.000, **c–d p=0.001**				
WOMAC stiffness score	3.82±0.98 4 (2–6)	2.30±0.84 2 (1–4)	2.86±0.88 3 (2–5)	2.11±0.71 2 (1–4)	<0.001^b^
p^bb^	**a–b p<0.001**, **a–c p=0.003**, **a–d p<0.001**, b–c p=0.092, b–d p=1.000, **c–d p=0.010**				
WOMAC function score	27.66±3.27 27.5 (21–35)	19.54±3.08 19 (15–31)	22.51±4.00 22 (16–30)	14.23±2.87 13 (11–24)	<0.001 ^b^
p^bb^	**a–b p<0.001**, **a–c p=0.002**, **a–d p<0.001**, b–c p=0.125, **b–d p<0.001**, **c–d p<0.001**				
WOMAC total score	37.87±3.23 39 (31–43)	24.57±2.76 25 (18–34)	31.16±4.10 31 (24–40)	19.23±3.58 19 (15–29)	<0.001^b^
p^bb^	**a–b p<0.001**, **a–c p=0.005**, **a–d p<0.001**, **b–c p=0.001**, **b–d p=0.016**, **c–d p<0.001**				

SD: standard deviation; TUG: timed up and go; WOMAC: Western Ontario and McMaster Universities Osteoarthritis Index.

a Fixed tibial insert and no patellar resurfacing.

b Fixed tibial insert and patellar resurfacing.

c Mobile tibial insert and no patellar resurfacing.

d Mobile tibial insert and patellar resurfacing.

b Kruskal-Wallis analysis of variance.

bb Kruskal-Wallis multiple comparison test.

p* Statistically significant level of difference between the four groups.

Bold values indicate statistically significant comparisons (p<0.05).

* Asterisks indicate statistically significant p-values (p<0.05).

For VAS scores, patients without patellar resurfacing had higher pain levels. Specifically, those with fixed inserts and no resurfacing had significantly higher scores than those with fixed inserts and resurfacing (p<0.001) and mobile inserts with resurfacing (p<0.001). Similarly, patients with mobile inserts and no resurfacing had higher VAS scores than those with fixed inserts and resurfacing (p<0.001) and mobile inserts with resurfacing (p=0.01).

For WOMAC pain scores, patients without patellar resurfacing had significantly higher scores. Those with fixed inserts and no resurfacing had higher scores than those with fixed inserts and resurfacing (p<0.001) and mobile inserts with resurfacing (p<0.001). Likewise, mobile inserts without resurfacing had higher WOMAC pain scores compared to fixed inserts with resurfacing (p<0.001) and mobile inserts with resurfacing (p=0.001).

For WOMAC stiffness scores, fixed inserts without resurfacing had significantly higher scores compared to all other groups (p<0.001 to p=0.003). Additionally, patients with mobile inserts and no resurfacing had higher stiffness scores than those with mobile inserts and resurfacing (p=0.01).

For WOMAC function scores, significant differences were found (p<0.001). Fixed inserts without resurfacing had the highest scores compared to all other groups (p<0.001). Patients with fixed inserts and resurfacing also had higher scores than those with mobile inserts and resurfacing (p<0.001).

For WOMAC total scores, fixed inserts without resurfacing had the highest scores, significantly exceeding those with fixed inserts and resurfacing (p<0.001), mobile inserts without resurfacing (p=0.005), and mobile inserts with resurfacing (p<0.001). Mobile inserts without resurfacing also had higher total WOMAC scores than fixed inserts with resurfacing (p=0.001) and mobile inserts with resurfacing (p<0.001). The lowest WOMAC total scores were observed in mobile inserts with patellar resurfacing.

According to Table 2, significant differences were found in TUG scores, flexion degrees, and extension limitation degrees among the groups (p<0.001), identified using the Kruskal-Wallis multiple comparison test.

**Table 2:** Comparison of timed up and gotest, flexion degree, and extension limitation degrees of patients in fixed insert and no patella resurfacing, fixed insert and patella resurfacing, mobile insert and no patella resurfacing, and mobile insert and patella resurfacing groups.

	Mean±SD; Median (Min–Max)				p*
	Fixed insert^a^ and no patella resurfacing (n=38)	Fixed insert^b^ and patella resurfacing (n=37)	Mobile insert^c^ and no patella resurfacing	Mobile insert^d^ and patella resurfacing (n=35)	
TUG	10.84±2.00 11 (8–15)	9.84±1.95 9 (7–14)	9.95±1.84 10 (8–15)	8.91±1.44 9 (7–13)	<0.001^b^
p^bb^	a–b p=0.168, a–c p=0.370, **a–d p<0.001**, b–c p=1.000, b–d p=0.245, c–d p=0.108				
Degree of flexion	103.24±8.87 105 (90–120)	104.73±8.07 105 (90–120)	110.86±8.41 110 (90–125)	112.57±7.80 115 (90–125)	<0.001^b^
p^bb^	a–b p=1.000, **a–c p=001**, **a–d p<0.001**, **b–c p=0.013**, **b–d p=0.001**, c–d p=1.000				
Extension limitation degree	8.29±3.66 8 (0–20)	8.03±3.27 10 (3–15)	4.76±3.03 5 (0–15)	4.11±3.19 5 (0–10)	<0.001^b^
p^bb^	a–b p=1.000, **a–c p<0.001**, **a–d p<0.001**, **b–c p<0.001**, **b–d p<0.001**, c-d p=1.000				

SD: standard deviation; TUG: timed up and go.

a Fixed tibial insert and no patellar resurfacing.

b Fixed tibial insert and patellar resurfacing.

c Mobile tibial insert and no patellar resurfacing.

d Mobile tibial insert and patellar resurfacing.

b Kruskal-Wallis analysis of variance.

bb Kruskal-Wallis multiple comparison test.

p* Statistically significant level of difference between the four groups.

Bold values indicate statistically significant comparisons (p<0.05).

* Asterisks indicate statistically significant p-values (p<0.05).

For TUG scores, patients with fixed inserts and no patellar resurfacing had significantly higher scores than those with mobile inserts and resurfacing (p<0.001), while no significant differences were found between other groups (p>0.05).

For flexion degrees, both fixed insert groups (with and without resurfacing) had significantly lower flexion compared to both mobile insert groups (p<0.001). No significant differences were observed between the two fixed insert groups or between the two mobile insert groups (p>0.05).

For extension limitation, patients with fixed inserts (both with and without resurfacing) had significantly higher limitations than both mobile insert groups (p<0.001). However, there were no significant differences within the fixed or mobile insert groups (p>0.05).

According to Table 3, no significant correlation was found between flexion degree and WOMAC Total scores in any group (p>0.05). In the fixed insert without resurfacing group, a positive correlation was observed between TUG scores and WOMAC Total scores (r=0.424, p<0.01), indicating that higher TUG scores were associated with higher WOMAC Total scores. In the fixed insert with resurfacing, mobile insert without resurfacing, and mobile insert with resurfacing groups, no significant correlation was found between flexion degree and WOMAC Total scores or TUG scores and WOMAC Total scores (p>0.05).

**Table 3:** Relationships between the degree of flexion and timed up and go scores and Western Ontario and McMaster Universities Osteoarthritis Index total scores in the groups.

		WOMAC total score			
		Fixed insert and no patella resurfacing	Fixed insert and patella resurfacing	Mobile insert and no patella resurfacing	Mobile insert and patella resurfacing groups
Degree of flexion	r*	-0.078	-0.133	-0.010	0.290
	p	0.640	0.434	0.952	0.091
TUG	r*	**0.424**	0.002	0.146	-0.115
	p	**0.008**	0.991	0.390	0.511

* Spearman's correlation coefficient.

TUG: timed up and go. Bold values indicate statistically significant comparisons (p<0.05).

According to Table 4, the Shapiro-Wilk test indicated that most outcome variables did not follow a normal distribution across the four groups (p<0.05 in several comparisons). Therefore, non-parametric tests such as the Kruskal-Wallis and Spearman's correlation analyses were appropriately used for group comparisons and correlation analysis, respectively.

**Table 4:** Shapiro-Wilk normality test results.

Variable	Fixed/no resurfacing (p)	Fixed/resurfacing (p)	Mobile/no resurfacing (p)	Mobile/resurfacing (p)
VAS	0.031	0.041	0.027	0.035
WOMAC Total	0.045	0.052	0.039	0.042
TUG	0.056	0.038	0.044	0.05
Flexion degree	0.021	0.047	0.036	0.04
Extension limitation	0.033	0.049	0.03	0.041

VAS: visual analog scale; TUG: timed up and go.

As shown in Table 5, a statistically significant positive correlation was observed between TUG scores and WOMAC total scores only in the fixed insert without patellar resurfacing group (r=0.424, p=0.008), suggesting that worse mobility was associated with greater self-reported disability in this subgroup. No significant correlations were identified between flexion degree and WOMAC total scores in any group.

**Table 5:** Correlation between functional scores.

Group	TUG vs. WOMAC (r, p)	Flexion vs. WOMAC (r, p)
Fixed/no resurfacing	0.424, p=0.008	-0.078, p=0.640
Fixed/resurfacing	0.002, p=0.991	-0.133, p=0.434
Mobile/no resurfacing	0.146, p=0.390	-0.010, p=0.952
Mobile/resurfacing	-0.115, p=0.511	0.290, p=0.091

TUG: timed up and go; WOMAC: Western Ontario and McMaster Universities Osteoarthritis Index.

## DISCUSSION

This study evaluated the effects of patellar resurfacing and tibial insert type (fixed vs. mobile) on clinical and functional outcomes in TKA. Our findings indicate that patellar resurfacing significantly improves pain relief (VAS) and functional outcomes (WOMAC, TUG, and flexion range). Additionally, mobile inserts provided better flexion range of motion and WOMAC functional scores compared to fixed inserts.

Comparative evaluation of mobile and fixed inserts in four different ways remains limited in the literature^13^. Capella et al.^14^ reported that mobile inserts enhance knee stability and flexion range, though direct comparisons with fixed inserts are scarce. While fixed inserts offer greater structural stability, they may limit joint mobility. In contrast, mobile inserts allow better load distribution and rotational movement, which may contribute to improved long-term function^15,16^. Our results align with the hypothesis that mobile-bearing designs facilitate more physiological kinematics, particularly in deep flexion activities^17^.

Our study is one of the few to comprehensively compare these two variables together. Patients with mobile inserts and patellar resurfacing had the lowest WOMAC scores, suggesting a synergistic benefit. This supports previous findings that patellar resurfacing improves pain scores and functional capacity, particularly in stair climbing, despite concerns about potential complications^18,19^. Recent meta-analyses have supported resurfacing, especially in patients with advanced patellofemoral degeneration^8,20^.

A notable finding was the positive correlation between TUG and WOMAC total scores in the fixed insert without patellar resurfacing group, suggesting that functional impairment is more pronounced in this subgroup. This further highlights the role of implant choice in optimizing mobility and quality of life. Interestingly, this correlation was not observed in the other three groups, possibly due to the compensatory biomechanical advantages of either patellar resurfacing or mobile insert use.

Considering the common association of knee osteoarthritis with obesity, the study's BMI restriction (<35) should be noted. Increased body weight exerts higher mechanical stress on the prosthesis, potentially accelerating wear and failure, especially in fixed inserts^21^. Patellar resurfacing may help alleviate these effects by improving patellofemoral load distribution, a finding supported by our results. Similarly, the broader load-sharing capacity of mobile inserts may contribute to prosthetic longevity^22,23^.

The biomechanical advantages of mobile inserts are well-documented. While fixed inserts provide joint stability by limiting mechanical movement, they may lead to localized stress concentrations, increasing wear risk^24^. Mobile inserts, in contrast, optimize load distribution and improve adaptability to rotational forces, which was reflected in better functional outcomes in our study^25^. Moreover, mobile designs may reduce polyethylene stress and micromotion, enhancing implant durability^26^. Additionally, patellar resurfacing enhances patellofemoral biomechanics by increasing contact surface alignment, further improving pain and function^27^.

### Limitations

This study has several limitations. Its retrospective nature may introduce selection and reporting bias. Although we used strict inclusion/exclusion criteria to reduce heterogeneity, unmeasured confounders such as postoperative rehabilitation compliance or surgeon preference may still have affected outcomes.

As a single-center study, the generalizability of the findings is limited. Postoperative rehabilitation intensity and patient-reported satisfaction were not recorded, which may influence the interpretation of functional outcomes. Additionally, long-term complications such as loosening, instability, or prosthetic survival were not evaluated. Another limitation is the relatively small subgroup sample sizes, which restricted our ability to perform robust multivariate regression analyses.

## CONCLUSION

This study suggests that mobile tibial inserts provide superior flexion range and functional outcomes compared to fixed inserts in TKA. Patellar resurfacing was associated with better pain relief and improved function, underscoring its role in optimizing surgical outcomes. The combination of patellar resurfacing and mobile-bearing tibial inserts yielded the most favorable results, indicating a potential synergistic effect. Future prospective, multicenter studies with long-term follow-up, standardized rehabilitation protocols, and radiographic monitoring are needed to confirm these findings and refine implant selection strategies in TKA.

## Data Availability

The datasets generated and/or analyzed during the current study are available from the corresponding author upon reasonable request.
